# Long-Read Sequencing Reveals Evolution and Acquisition of Antimicrobial Resistance and Virulence Genes in *Salmonella enterica*

**DOI:** 10.3389/fmicb.2021.777817

**Published:** 2021-11-19

**Authors:** Cong Li, Gregory H. Tyson, Chih-Hao Hsu, Lucas Harrison, Errol Strain, Thu-Thuy Tran, Glenn E. Tillman, Uday Dessai, Patrick F. McDermott, Shaohua Zhao

**Affiliations:** ^1^Center for Veterinary Medicine, United States Food and Drug Administration, Laurel, MD, United States; ^2^Food Safety and Inspection Service, United States Department of Agriculture, Athens, GA, United States; ^3^Food Safety and Inspection Service, United States Department of Agriculture, Washington, DC, United States

**Keywords:** *Salmonella*, multidrug resistance (MDR), plasmid, *Salmonella* genomic island (SGI), *Salmonella* pathogenicity island (SPI)

## Abstract

*Salmonella enterica* is a significant and phylogenetically diverse zoonotic pathogen. To understand its genomic heterogeneity and antimicrobial resistance, we performed long-read sequencing on *Salmonella* isolated from retail meats and food animals. A collection of 134 multidrug-resistant isolates belonging to 33 serotypes were subjected to PacBio sequencing. One major locus of diversity among these isolates was the presence and orientation of *Salmonella* pathogenic islands (SPI), which varied across different serotypes but were largely conserved within individual serotypes. We also identified insertion of an IncQ resistance plasmid into the chromosome of fourteen strains of serotype I 4,[5],12:i:– and the *Salmonella* genomic island 1 (SGI-1) in five serotypes. The presence of various SPIs, SGI-1 and integrated plasmids contributed significantly to the genomic variability and resulted in chromosomal resistance in 55.2% (74/134) of the study isolates. A total of 93.3% (125/134) of isolates carried at least one plasmid, with isolates carrying up to seven plasmids. We closed 233 plasmid sequences of thirteen replicon types, along with twelve hybrid plasmids. Some associations between *Salmonella* isolate source, serotype, and plasmid type were seen. For instance, IncX plasmids were more common in serotype Kentucky from retail chicken. Plasmids IncC and IncHI had on average more than five antimicrobial resistance genes, whereas in IncX, it was less than one per plasmid. Overall, 60% of multidrug resistance (MDR) strains that carried >3 AMR genes also carried >3 heavy metal resistance genes, raising the possibility of co-selection of antimicrobial resistance in the presence of heavy metals. We also found nine isolates representing four serotypes that carried virulence plasmids with the *spv* operon. Together, these data demonstrate the power of long-read sequencing to reveal genomic arrangements and integrated plasmids with a high level of resolution for tracking and comparing resistant strains from different sources. Additionally, the findings from this study will help expand the reference set of closed *Salmonella* genomes that can be used to improve genome assembly from short-read data commonly used in One Health antimicrobial resistance surveillance.

## Introduction

*Salmonella enterica* is an important zoonotic pathogen that causes over one million illnesses in the United States each year (Scallan et al., 2011). *S. enterica* are classically subdivided into serotypes and over 2,600 serotypes have been identified thus far. While many serotypes may be capable of causing infections in humans and animals, a limited number of serotypes cause most human infections in the United States. Recent advancements in whole genome sequencing (WGS) offer a unique opportunity to dissect and investigate *Salmonella* serotypes at the nucleotide level and to further our understanding about notable evolutionary changes. The main features associated with *S. enterica* evolution include acquisition and recombination of mobile genetic elements such as genomic islands, transposons, integrons, and plasmids, among others (Partridge et al., 2018). An in-depth analysis of these features will help us to understand drivers of resistance, host and environmental adaptations, and sources of resistant *Salmonella* infections.

While most *Salmonella* infections are self-limiting, serious infections can require antimicrobial therapy (Tack et al., 2020). Antimicrobial resistance (AMR) can compromise therapy, increase healthcare costs, and cost lives (Abebe et al., 2020). In *Salmonella*, AMR is typically attained by horizontal acquisition of antimicrobial resistance genes (ARGs), although chromosomal mutations also play a role (McDermott et al., 2016).

One way that *Salmonella* strains acquire ARGs is through acquisition of plasmids (Emond-Rheault et al., 2020). Plasmids carry not only ARGs, but also heavy metal and disinfectant resistance genes, which may contribute to co-selection for AMR (Vijayakumar and Sandle, 2019). The types of plasmids that *Salmonella* carry can vary considerably, as they may include species-specific non-conjugative plasmids, or conjugative plasmids found widely among Enterobacterales (Redondo-Salvo et al., 2020). Some plasmid types are highly associated with specific serotypes and sources (Zhao et al., 2020), thus plasmids provide important information for outbreak investigations and AMR source attribution. Traditionally, incompatibility plasmid types have been used to assign plasmids into different groups based on plasmid replication machinery (Carattoli, 2013). This approach does not account for all plasmid types and it is often unclear which replication machinery is dominant, especially in hybrid plasmids arising from recombination (Hsu et al., 2019).

Characterization of plasmids and other resistance elements in *Salmonella* has been studied extensively by WGS. The use of short-read sequencing in conjunction with programs such as PlasmidSpades, PLACnet, or others have helped expand analyses of genomes derived from short-read sequencing data (de Toro et al., 2014; Antipov et al., 2016). There have been relatively few large-scale, long-read sequencing studies, which can yield more complete genomic information with higher resolution.

Aside from plasmids, ARGs also are commonly carried by chromosomally encoded *Salmonella* genomic islands (SGIs). SGI-1 was first reported in *S.* Typhimurium DT104 in 2001. It contained a 27 kb backbone plus a 15 kb complex with a class 1 integron, with ARGs conferring resistance to five antimicrobial classes (Boyd et al., 2000). Different variants of SGI-1 have been described, with a diversity of ARG alleles in multidrug resistance (MDR) regions (Hall, 2010). Additional SGIs, including SGI-0, SGI-2, SGI-3, and SGI-4, have been identified based on genomic structure and resistance gene contents. Both SGI-0 and SGI-2 are in the same location as SGI-1 and shared the SGI-1 backbone sequence (Levings et al., 2008; de Curraize et al., 2020). SGI-3 and SGI-4 were initially described as distinct SGIs, but they are in the same chromosomal location, have the same sequence backbone structure and are considered the same SGI (Arai et al., 2019; Branchu et al., 2019). SGI-4 did not carry AMR genes, instead it carried 24 heavy metal resistant genes (HMRGs) (Arai et al., 2019). Together the acquisition of AMR determinants, mobile genetic elements contribute to the genomic diversity found in *Salmonella.*

*Salmonella* pathogenic islands (SPIs) play a pivotal role in *Salmonella* virulence (Hensel, 2004; Rychlik et al., 2009). There are 24 known SPIs and several are associated with particular mechanisms of virulence (Cheng et al., 2019). SPI-1 and SPI-2, which encode type III secretion systems, are conserved across *Salmonella*, along with SPIs 3-6, SPI-9, and SPI-11 (Jennings et al., 2017; Lou et al., 2019; Zhao et al., 2020). Most other SPIs are variable across serotypes and may account for differences in virulence among *Salmonella* serotypes (Zhao et al., 2020). Thus, the presence and organization of these SPIs are important to understanding the evolution and pathogenicity in *Salmonella*.

The history of *Salmonella* epidemiology has relied on various features to categorize strains. Following biochemical profiling, serotyping has long been the basis of *Salmonella* strain typing and tracking. Later, plasmid profiling by electrophoresis and pulsed field gel electrophoresis (PFGE) were used. For AMR monitoring in national programs such as the United States National Antimicrobial Resistance Monitoring System (NARMS), minimum inhibitory concentration (MIC) testing followed by multiplex PCR and conjugation assays have been commonly used to track phenotypic and genotypic resistance. With the advent of affordable WGS, surveillance of AMR *Salmonella* and other pathogens can now be done routinely using short read DNA sequencing chemistries. While this provides a comprehensive picture of strain relatedness and gene carriage, closed genomes are needed to reveal the detailed gene arrangements and structural changes. In this report, we describe the use of PacBio long-read sequencing to characterize 134 isolates, representing 33 *Salmonella* serotypes, isolated from raw meats and food animals. This study helped us to elucidate the genomic structure and location of virulence and resistance genes, their colocation on mobile DNA elements, and how these traits relate to *Salmonella* evolution. We also proposed a new approach to simplify naming of SGIs based on their genomic position.

## Materials and Methods

### Isolate Sources and PacBio Sequencing

One hundred thirty-four isolates, representing 33 serotypes, were collected as part of routine surveillance by the NARMS. The sources of these isolates were chicken, turkey, beef, and pork products as well as cecal/gut samples collected at slaughter from swine, turkey, cattle, and chicken from 2016–2018 across 31 different states. Isolates were selected for Pacific Biosciences (PacBio) long-read sequencing to represent diverse resistance patterns including three pan-susceptible isolates, diverse serotypes and different NARMS sources (Supplementary Table 1).

For long-read sequencing, DNA libraries were prepared using a 10 kb template preparation protocol with SMRTbell template prep kit v 1.0. Sequencing was performed using Pacific Biosciences technology on the Sequel platform with sequencing kit 3.0, as described previously (Tate et al., 2021).

Sequencing data are available in BioProject PRJNA292661. Isolate-level accession numbers are listed in Supplementary Table 1.

### Resistance Gene and Plasmid Identification

Antimicrobial resistance genes, biocide resistance genes, and HMRGs were identified with AMRFinder Plus version 3.8 (Feldgarden et al., 2019). The AMRFinder Plus virulence genes and ARGs outside the AMRFinder core genes were not reported, due to their limited relevance to this *Salmonella* study.

To identify plasmid replicon sequences, we used PlasmidFinder with cutoffs of 90% identity and 60% length (Carattoli et al., 2014). The sequence of the *spvRABCD* operon was extracted from the plasmid pOU1115 carried by a *S.* Dublin strain (Accession DQ115388). A local blastn analysis with the same cutoffs was performed to identify the presence of this *spv* operon.

Integrated plasmids were also identified by a similar approach, with PlasmidFinder being used to identify replicons. In some cases, blastn analysis was conducted to identify similarity with known plasmids (Table 2).

### *Salmonella* Pathogenic Islands and *Salmonella* Genomic Island Identification

Sequences of 24 SPIs were downloaded from GenBank to a local database (Fookes et al., 2011; Hayward et al., 2014; Cheng et al., 2019; Hsu et al., 2019). The size of the SPIs ranged from 1.7 to 133.3 kb, encoding 1–21 virulence genes. Due to the variable length and gene content of SPIs, specific SPIs were identified as present if any of its associated virulence genes in the database developed previously (Blondel et al., 2009; Suez et al., 2013; Zhao et al., 2020) were identified with 85% identity and at least 70% length by blastn v.2.7 and anchored by blasting chromosome sequences with the reference SPIs sequence using 85% identity and at least 10% length. SPI-8 and SPI-13 have the same genomic location adjacent to tRNA-*pheV* (Espinoza et al., 2017), and seven isolates from four serovars carried 17 kb (70%) SPI-13 sequence but without its virulence genes were given the name of SPI-13^∗^ in this paper.

SGI-1 and potential variant sequences were initially identified by blast with 85% identity and 70% length to 47 kb of reference SGI-1 sequence from *S.* Typhimurium DT104 (Boyd et al., 2000). Further analysis to identify additional SGI-1 sequences involved identifying insertions between *yid*Y(5′) and *thd*F(3′), with seven additional SGI variants identified. The existence of SGI-4 was discovered using a blast query of chromosomal sequences against SGI-4 reference (MN730129.1) with 85% coverage and 70% homology cutoffs. The additional SGI-4 was discovered by aligning the N16S319 and other *S.* Alachua without resistance genes. The other islands with ARGs and/or HMRGs were discovered using comparisons to chromosomes within the same serotypes in isolates lacking ARGs and HMRGs.

### Phylogenetic Tree

The program KSNP3.0 was used to generate Single Nucleotide Polymorphisms (SNPs) from a subset of 44 complete chromosomes to represent all 33 serotypes and cases of chromosomal heterogeneity within serotypes (Gardner et al., 2015). Prior to SNP generation, the kmer size was chosen by Kchooser included in KSNP3.0 (Gardner et al., 2015). The maximum likelihood phylogeny tree was constructed by MEGA7.0 with 250 bootstraps. The clade including *S.* Montevideo and *S.* Schwarzengrund was placed at the root based on established literature (Worley et al., 2018).

## Results

### Presence of *Salmonella* Pathogenic Islands and Arrangement in the Chromosome

Assembly of long-read sequences produced circular closed chromosomes in 116 of the 134 *Salmonella* isolates, ranging in size from 4,492,868 bp in *S.* Bredeney to 5,073,615 bp in *S.* I 4,[5],12:i:-. Nine isolates showed genomic size ¿5,000,000 bp, with six of them I 4,[5],12:i:-, two *S*. Agona and one *S*. Typhimurium (Supplementary Table 1). Our data showed that additional accessary genomic elements, such as phage, integration of plasmids in chromosome or SGI contributed to the larger genomes.

**FIGURE 1:**
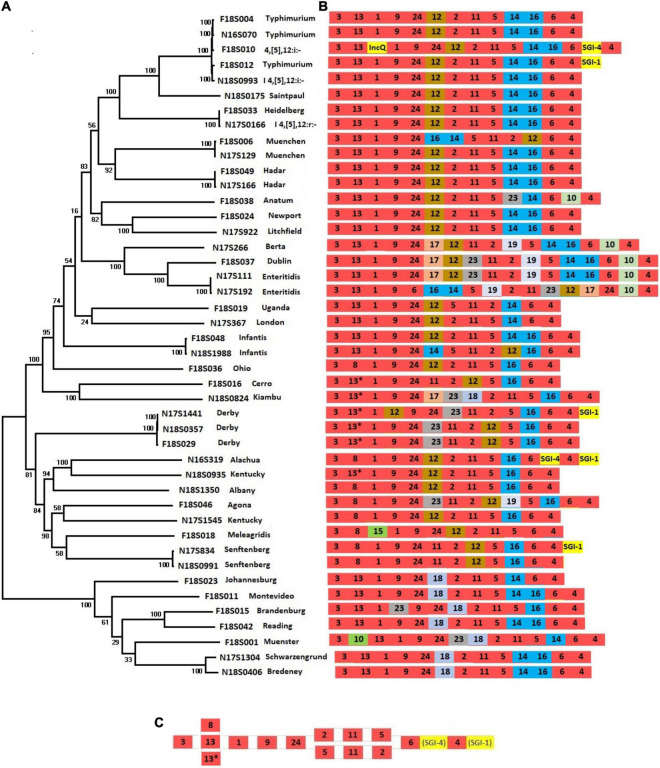
Presence of SPIs in different *Salmonella* serotypes. **(A)** A phylogenetic tree was constructed based on chromosomal SNPs of each of the isolates. **(B)** SPI arrangements are listed with representative sequences of each serotype. Red indicates that SPIs are conserved in all serotypes, yellow indicates insertions of SGIs or plasmids. Other colors represent SPIs whose presence are variable among different serotypes/isolates. **(C)** The conserved structure across all serotypes is shown.

*Salmonella* pathogenic islands contain a variety of genes that contribute to *Salmonella* virulence as part of *Salmonella* serotype evolution (Marcus et al., 2000). To assess the complement of SPIs that contribute to the diversity of chromosomal sequences from different serotypes, we constructed a phylogenetic tree using SNPs across 44 chromosomes from 34 serotypes (Figure 1A). This tree reflects the phylogenetic relationships among different *Salmonella* serotypes and is reflective of their entire genomic content.

The SPIs 1-6, SPI-9, SPI-11, and SPI-24 were present in all isolates (Figure 1B and Supplementary Table 1). SPI-24 was previously called a CS54 (Sabbagh et al., 2010), but later renamed as SPI-24 (Cheng et al., 2019). Each of these conserved SPIs were largely in conserved locations as well, except for large inversions encompassing multiple SPIs in serotypes Infantis, Muenchen, Typhimurium, and Enteritidis (Figures 1B,C). Three SPIs, including SPI-20, SPI-21, and SPI-22, were not identified in our isolate collection.

*Salmonella* pathogenic islands were largely conserved within serotypes but showed varying degrees of diversity between serotypes, consistent with previous reports (Hsu et al., 2019; Zhao et al., 2020). As expected from the close relationship between *S.* Typhimurium and its monophasic variant *S.* I 4,[5],12:i:- (Ido et al., 2014), their complement of SPIs was identical (Figure 1). In fact, the large clade of related serotypes from Typhimurium to Litchfield all had almost identical combinations of SPIs (Figure 1B). Only a few serotypes displayed SPI variability among their strains. Interestingly, none of the SPIs were serotype-specific, as each of the 21 identified SPIs were distributed among multiple serotypes. Large inversions were observed within some serotypes. For example, in one of two *S.* Enteritidis strains, a large fragment from SPI-6 to SPI-17 was inverted, and in one of the *S.* Infantis strains the region between SPI-12 to SPI-14 was inverted (Figure 1B).

### Acquisition of Genomic Islands and Associated Antimicrobial Resistance Genes and Heavy Metal Resistant Genes

In this study, SGI-1 and SGI-1 variants were found among 12 strains including serotypes Typhimurium (*n* = 5), Senftenberg (*n* = 2), Derby (*n* = 3), Saintpaul (*n* = 1), and Alachua (*n* = 1) (Table 1). The location of insertion of SGI-1 was consistent across these different serotypes, present between SPIs 4 and 3 in the assembled chromosomes. The backbone and ARGs in SGIs varied greatly in different strains. All five *S.* Typhimurium SGI-1 carried similar backbone structure and ARGs, *aadA2-qacEdelta-sul1-floR-tet(G)-blaCARB-2-qacEdelta* as previously reported (GenBank accession number AF261825), except that in N17S016, whose SGI was 9 kb shorter and contained only *sul1* and *bla*_CARB–__2_ ARGs.

**TABLE 1:** Putative *Salmonella* genomic islands in the genomes of *Salmonella* isolates.

Strain ID	Serotype	SGI	Size	Accession	Position	Top non-*Salmonella* hit of organism and coverage, identity	Accession#	Resistance genes
F18S012 F18S028 F18S031 N16S132	Typhimurium	SGI-1	47,722	OK209931	SPI-3, SPI-4	*Proteus mirabilis* (89%, 100%)	KJ186152.1	*qacEdelta1 bla*_CARB–__2_ *tet(G) floR sul1 qacEdelta1 aadA2*
N17S016	Typhimurium	SGI-1	38,450	OK209935	SPI-3, SPI-4	*Proteus mirabilis* (89%, 100%)	KJ186152.1	*sul1 qacEdelta1 bla*_CARB–__2_
N17S1441 N18S0357	Derby	SGI-1	43,948	OK209937	SPI-3, SPI-4	*Proteus mirabilis* (89%, 100%)	MK422178.1	*tet(A) merR merT merP merC sul1 qacEdelta1*
N18S0789	Derby	SGI-1	44,852	OK209939	SPI-3, SPI-4	*Proteus mirabilis* (64%, 100%)	MK422178.1	*tet(A) merR merT merP merC sul1 qacEdelta1 aadA2 dfrA12*
N16S319	Alachua	SGI-1	63,305	OK209933	SPI-3, SPI-4	Proteus mirabilis (93%, 100%)	KJ439039.1	*merE merD merF merP merT merR sul1 qacEdelta1 aadA1 tet(A) aph(6)-Id aph(3*″*)-Ib*
F18S026	Senftenberg	SGI-1	47,273	OK209932	SPI-3, SPI-4	*Proteus mirabilis* (86%, 100%)	KJ439039.1	*merE merD merF merP merT merR sul1 qacEdelta1 aadA1 tet(A) aph(6)-Id aph(3*″*)-Ib aph(3*′*)-Ia*
N17S834	Senftenberg	SGI-1	117,891	OK209936	SPI-3, SPI-4	*Citrobacter koseri* (66%, 89%)	CP026697.1	*merP merT merR sul1 qacEdelta1 dfrA5 tet(A) aph(6)-Id aph(3*″*)-Ib*
N18S0175	Saintpaul	SGI-1	29,745	OK209938	SPI-3, SPI-4	*Proteus mirabilis* (97%, 100%)	KJ439039.1	*bla*_TEM–__1_ *sul1 qacE aadA2 ant(2*″*)-Ia*
F18S010 F18S014 F18S032 F18S040 F18S043 F16S144 N17S056 N17S1466 F18S030 N18S0173	I 4,[5],12:i:-	SGI-4	81,780	MN730129.1	SPI-4, SPI-6	*Citrobacter* sp. (45%, 97%)	CP056647.1	*pcoS pcoR pcoD pcoC pcoA silP silA silB silF silC silR silS silE arsC arsBarsA arsD arsR*
N16S319	Alachua	SGI-4	∼85,000	OK209934	SPI-4, SPI-6	*Enterobacter hormaechei* (97%, 100%)	CP042551.1	*silE silS silR silC silF silB silA silP pcoA pcoB pcoC pcoD pcoR pcoS pcoE*

**TABLE 2:** Putative integrated plasmids in *Salmonella* isolates.

ID	Serotype	Estimated size of plasmid on chromosome	Location of insertion	AMR and HMR on inserted plasmid	Match with reference plasmid (accession No)	Plasmid types
F18S002 F18S010 F18S014 F18S032 F18S040 F18S043 F18S045 N16S144 N17S107 N17S380 N17S1466 N18S0173	I 4,[5],12:i:-	16 kb	SPI-1, SPI-9	*tet(B) merR merT merP merC sul2 aph(3*″*)-Ib aph(6)-Id bla*_TEM–__1_	pHCM1 (CP029645.1)	IncQ
N17S146	I 4,[5],12:i:-	12 kb	SPI-1, SPI-9	*sul2 merC merP merT merR tet(B)*	pHCM1 (CP029645.1)	IncQ
F18S001	Muenster	31.6 kb	SPI-5, SPI14	*silE silS silR silC silF silB silA silP pcoA pcoB pcoC pcoD pcoR pcoS pcoE*	pF18S044-1 (ready for submission)	IncHI2 IncHI2A
F18S023	Johannesburg	31.6 kb	SPI-6, SPI14	*SilE silS silR silC silF silB silA silP pcoA pcoB pcoC pcoD pcoR pcoS pcoE*	pF18S044-1 (ready for submission)	IncHI2 IncHI2A
N17S0834 F18S026 N18S0991	Senftenberg	31.6 kb	SPI-3, SPI-8	*SilE silS silR silC silF silB silA silP pcoA pcoB pcoC pcoD pcoR pcoS pcoE*	pF18S044-1 (ready for submission)	IncHI2 IncHI2A
N18S0017	Agona	31.6 kb	SPI-3, SPI-8	*SilE silS silR silC silF silB silA silP pcoA pcoB pcoC pcoD pcoR pcoS pcoE*	pF18S044-1 (ready for submission)	IncHI2 IncHI2A
F18S033	Heidelberg	31.6 kb	SPI-5, SPI14	*SilE silS silR silC silF silB silA silP pcoA pcoB pcoC pcoD pcoR pcoS pcoE*	pF18S044-1 (ready for submission)	IncHI2 IncHI2A
N17S1304 N18S1602	Schwarzengrund	31.6 kb	SPI-3, SPI-13	*silE silS silR silC silF silB silA silP pcoA pcoB pcoC pcoD pcoR pcoS pcoE*	pF18S044-1 (ready for submission)	IncHI2 IncHI2A
F18S034	Derby	77.3 kb	SPI-1, SPI-3	*SilE silS silR silC silF silB silA silP pcoA pcoB pcoC pcoD pcoR pcoS pcoE tet(A) merR merT merP bleO*	pF18S029-1 (ready for submission)	IncHI2 IncHI2A
F18S013	Typhimurium	71 kb	SPI-5, SPI14	*bla*_CMY–__2_	pF18S007-1 (ready for submission)	IncI
N16S098	Heidelberg	71 kb	SPI-6, SPI-16	*bla*_CMY–__2_	pF18S007-1 (ready for submission)	IncI
F18S033	Heidelberg	54 kb	SPI-4, SPI-6	*qacEdelta1 cmlA5 ant(2*″*)-Ia tet(A) aph(6)-Id aph(3*″*)-Ib sul2*	p24358-2 (CP051360.1)	IncC
N18S0597 N18S1595 N18S2170	Typhimurium	85 kb	SPI-1, SPI-9	*terW terZ terD tet(C) aadA1 aac(3)-VIa qacEdelta1 sul1 merE merD merA merT merR silE silS silR silC silF silB silA silP*	pSDC-F2_12BHI2 (MH287085.1)	IncHI2 IncHI2A
N18S0981	Typhimurium	76.4 kb	SPI-1, SPI-9	*terW terZ terD tet(C) aadA1 aac(3)-VIa qacEdelta1 sul1 merE merD merA merT merR silE silS silR silC silF silB silA silP*	pSDC-F2_12BHI2 (MH287085.1)	IncHI2 IncHI2A
N18S0597 N17S0520 N18S0666 N18S0981 N18S2170	Typhimurium	107 kb	SPI-6, SPI-16	*merR tet(A) sul2*	pN18S1634-2	IncC
N17S0520 N18S0666 N18S0981 N18S2170	Typhimurium	83.5 kb	SPI-6, SPI-16	*merR tet(A) sul2*	pN18S1634-2	IncC
N18S1595	Typhimurium	56 kb	SPI-6, SPI-16	*merR tet(A) sul2*	pF18S004	Unknown - not closed
N17S0166	I 4,[5],12:r:-	158 kb	SPI-1, SPI-9	*pcoS mcr-9.1 arsC aadA1 aac(3)-VIa qacEdelta sul1*	pN53053 (CP049311.1)	IncHI2 IncHI2A
N18S0736	I. 4,[5],12:r:-	38 kb	SPI-1, SPI-9	*sul1, qacEdelta1, aac(3)-VIa, aadA1, arsR*	pN53053 (CP049311.1)	IncHI2 IncHI2A
F18S004	Typhimurium	21 kb	SPI-6, SPI-9	*tetW tetZ tetD*	pF18S044-1	IncHI2 IncHI2A
F18S013	Typhimurium	3.8 kb	SPI-5, SPI-14	*bla*_CMY–__2_	pF18S003-1/pF18S007-1/pN16S065-2	IncC/IncI/IncB/O/K/Z
N16S021 N16S070 N16S089 N16S098 N16S189 N16S214 N18S0645 18S1677 N18S2188	Typhimurium	3.8 kb	SPI-9, SPI-12	*bla*_CMY–__2_	pF18S003-1/pF18S007-1/pN16S065-3	IncC/IncI/IncB/O/K/Z
N18S0406	Bredeney	3.8 kb	SPI-3, SPI-13	*bla*_CMY–__2_	pF18S003-1/pF18S007-1/pN16S065-10	IncC/IncI/IncB/O/K/Z
F18S049 N17S1270 N18S1943 N18S2154	Hadar	15.0–19.3 kb	SPI-2, SPI-12	*aph(3*″*)-Ib aph(6)-Id tet(A)*	pH1038-142(KJ484634.1)	IncN IncFII
N18S2042	Infantis	91 kb	SPI-2, SPI-12	*tet(A) merR merT merP merC bla*_CTX–M–__65_	pN16S024 (CP052840.1)	IncHI2

The three *S.* Derby strains carried similar SGI-1 backbone structure (43.9–44.9 kb) as the *S.* Typhimurium strain, and all three carried a similar resistance gene cassette (Table 1), with 2 to 4 ARGs and 4 mercury resistance genes.

The remaining four SGI-1 sequences varied greatly in size, from 29.7 kb in a *S.* Saintpaul strain to 117.9 kb in a *S.* Senftenberg strain (Table 1). The structures and resistance gene content varied considerably, with 5–6 ARGs and 0–6 mercury resistance genes (Table 1). Interestingly, although the top NCBI blast hits were for *Salmonella*, all SGI-1s are found to be homologous or partially homologous (Table 1) to the genomic islands on *Proteus*. The *S.* Senftenberg SGI-1 was particularly notable since it appeared to have a hybrid origin with 51 kb aligning with other SGI-1 sequences and a 67 kb homology with that of *Citrobacter* (Table 1).

SGI-4 (MN730129.1) was found in ten *S.* I 4,[5],12:i:- strains and was located between SPI-4 and SPI-6 (Table 1). As previously reported, it did not carry ARGs and only carried HMRGs *pco*, *sil*, and *ars* encoding for resistance to copper, silver, and arsenic, respectively (Bearson et al., 2020).

An additional potential estimated 84 kb genomic island (Table 1) was found in *S.* Alachua (Table 1). It has some homology to SGI-4 with 95% identity and 40% length. Although not experimentally validated as an SGI, it has many similarities to SGI-4 including its location between SPI-4 and SPI-6 and presence of the *sil* and *pco* HMRGs. This region also has genes related to conjugative transfer and partitioning, indicating that it is likely a mobile element.

### Integration of Plasmids Into the Chromosome and Associated Antimicrobial Resistance Genes and Heavy Metal Resistant Genes

**FIGURE 2:**
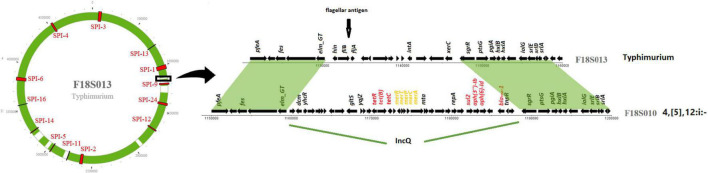
IncQ plasmid integrated into *Salmonella* I 4,[5],12:i:- chromosome. The circle on the left represents a chromosome structure of a *S.* Typhimurium strain F18S013 with SPIs (in red) distributed on the chromosome. The green area was the sequence homologous to a *Salmonella* I 4,[5],12:i:- strain F18S010. The gray area shows differences between the two strains. The detailed comparison of the genomic structure of the two strains on the right is from the area with black box on the chromosome.

IncQ plasmid replicons were detected in chromosomal sequences of fourteen *S.* I 4,[5],12:i:- isolates (Table 2). Interestingly, the location of IncQ plasmids was in the same region as the *fljB* gene encoding the phase two H antigen in *S.* Typhimurium, and the *fljB* region missing in *S.* I 4,[5],12:i:- (Figure 2). This finding suggests a plasmid to chromosome recombination event that transformed strains from serotype Typhimurium to I 4,[5],12:i:-. In each of the *S.* I 4,[5],12:i:- genomes in this study the recombination event had introduced *sul2* and in all but one isolate *tetB*, *bla*_TEM–__1_ and *aph(3*″*)-Ib/aph(6)-Id* were introduced.

Another similar recombination event was detected in strain N17S166 of serotype I.4,[5],12:r:-. In this isolate a fragment of 152 kb from a IncHI plasmid (pN17S1352-1) carried *mcr-9.1*, *aadA1*, *aac(3)-VIa*, *sul1* and two HMRGs, *pcoS* and *arsC*, integrated into the chromosome between SPI-1 and SPI-9 in the same region where *S.* Heidelberg carried a *flj*B gene (Table 2).

There are additional examples of ARGs or HMRGs in the chromosome that may have resulted from plasmid integrations (Table 2). For three *S.* Typhimurium strains from retail chicken (N18S0597, N18S1595, and N18S2170), there are two chromosomal regions with ARGs and HMRGs. The first is an up to 107 kb region located between SPI-6 and SPI-16 with *merR*, *tet*(A), and *sul2*, and can be traced back to an IncC plasmid pN18S1634-2. The second region is about 85 kb and has over 70% alignment to a previously published IncHI plasmid (nucleotide accession MH287085.1). This 85 kb region carries five ARGs and 16 HMRGs. Another common insertion included a 31.6 kb element with silver and copper resistance genes. This insertion was found in the chromosomes of nine isolates, including serotypes Muenster, Johannesburg, Senftenberg, Heidelberg, Schwarzengrund, and Agona, and across multiple animal sources (Table 2). This insertion has high sequence homology with the IncHI plasmid pF18S044-1 (Table 2). These findings reveal how plasmids or their remnants can contribute to the chromosomal acquisition of ARGs and HMRGs.

The most common serotypes with chromosomal ARGs were Typhimurium (20/24 isolates), I 4,[5],12:i:- (15/16), Agona (10/10), and Heidelberg (7/7). In contrast, serotypes Kentucky (0/11) and Reading (0/6) did not have any chromosomal ARGs. Among the nine isolates that did not carry any plasmids, no chromosomal ARGs were found in three isolates (N17S192, N17S312, and N18S1429) which were pan-susceptible to all antimicrobials tested, *fosA* was found in two (N18S0476 and N18S0722), and the remaining four (N17S107, N17S834, N18S0173, and N18S2170) carried multiple chromosomal ARGs (Supplementary Table 1). These isolates encompassed seven different serotypes.

Chromosomal HMRGs were found in 51 of 134 isolates, including genes encoding resistance to copper, silver, mercury, and arsenic. These comprised 22 turkey, 14 swine, 11 chicken, and 4 cattle isolates. Thirty-nine of the fifty-one isolates with more than three chromosomal HMRGs also had chromosomal ARGs. In many cases, HMRGs and ARGs were physically linked (Table 2).

Together these findings among 134 *Salmonella* genomes show that the maintenance and spread of chromosomal ARGs and HMRGs in *Salmonella* is accomplished through a complex interplay of genomic islands and integrated plasmids. Further work will be needed to understand whether acquisition of these genes is specifically selected for by exposure to heavy metals and/or antimicrobials or connected to other survival and fitness challenges faced by *Salmonella*.

### Plasmid Types and Association With Resistance Genes, Sources, and Serotypes

**FIGURE 3:**
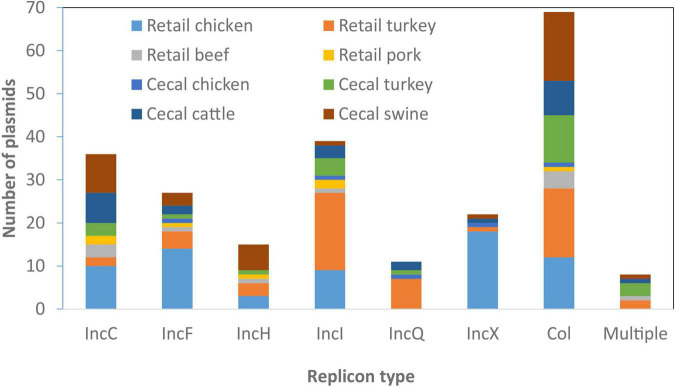
Plasmid types present across different food animal sources. Replicon information was based on analysis from PlasmidFinder. Only the most common plasmid types are represented in the figure.

From the 134 *Salmonella* isolates we developed high-quality sequences for 285 plasmids, 245 of which we fully resolved into circular structures. On average there were two plasmids per isolate, although seven isolates had no plasmids and one had as many as seven plasmids, based on closed, circular sequences (Supplementary Table 1). We sought to identify the AMR and HMRGs on these plasmids and evaluated if any plasmids had animal source or serotype-specificity. Although some plasmid types were most common in certain sources, most were widely distributed across all animal sources (Figure 3); some association between plasmid type and serotype was also noticed (Table 3).

**TABLE 3:** Association of plasmid types with antimicrobial resistance genes (ARGs) and heavy metal resistant genes (HMRGs).

Plasmid types	Total number	Avg. size in kb (range)	Most common serotype (number)	Avg. ARGs (range)	Avg. HMRGs (range)
IncA	2	133 (90–176)	Heidelberg, I. 4,[5],12:i:- (1)	5.5 (5–6)	2.5 (0–5)
IncB/O/K/Z	1	115	Kentucky	4	4
IncC	36	142 (52–232)	Typhimurium (11)	6.6 (0–13)	3.8 (0–8)
IncF	23	112 (16–164)	Kentucky (9)	2.1 (0–6)	0.8 (0–7)
IncH	15	270 (145–354)	Kentucky (3)	5.4 (1–14)	13.9 (3–22)
IncI	39	99 (53–125)	Kentucky, I. 4,[5],12:i:- (7)	1.6 (0–4)	0.7 (0–15)
IncN	3	57 (43–71)	Enteritidis, Heidelberg, I 4,[5],12:i:-	3 (1–5)	0
IncP	1	18	I 4,[5],12:i:-	2	0
IncQ	11	11 (8–12)	Reading (5)	3.3 (1–4)	0
IncR	1	70	Muenster	10	0
IncX	22	42 (31–53)	Kentucky (9)	0.4 (0–6)	0
IncY	1	92	I. 4,[5],12:i:-	0	0
Col	69	5 (2–15)	Typhimurium (11)	0.3 (0–2)	0
Phage-like	1	91	Typhimurium	0	0
Combination	12	237 (75–389)	Infantis (4)	6.3 (0–12)	6.3 (0–18)
Unknown (no replicon)	28	18 (1–186)	Typhimurium (5)	0.6 (0–6)	0.5 (0–14)
Chromosome	134	4.8 Mb (4.5–5.1 Mb)	N/A	1.7 (0–7)	7.3 (0–25)

#### IncC

Sizes of the 36 IncC plasmids varied considerably, from 52 to 232 kb, and were found among all animal sources (Table 3). These plasmids were found among 11 different serotypes, with the most prevalent being *S.* Typhimurium. Some common resistance patterns emerged, and 34 of the plasmids had *sul2* and *tetA*, and 22 had all of *bla*_CMY–__2_, *floR*, and *aph(6)-Id/aph(3*″*)-Ib*. Twelve of the isolates also had at least one *qac* gene and 29 had at least one mercury resistance gene.

#### IncHI

There were 15 IncHI plasmids identified among twelve different serotypes, present in all food animal sources, with swine being most common (7/15, 47%). All were large plasmids of 145–354 kb and possessed from one to fourteen ARGs. Seven also had the *qacE* biocide resistance gene, and all had at least one metal resistance operon, including those conferring resistance to silver, copper, mercury, and arsenic.

#### IncI

There were 39 IncI plasmids in our collection of 134 genomes, from 19 different serotypes, with at least one ARG found in 33 of the plasmids. They ranged in size from 62 to 125 kb and were found from all four food animal sources. The most common ARG was the clinically relevant *bla*_CMY–__2_, which was found in 15 isolates, including six *S.* Kentucky from retail chicken. Only two of the plasmids had HMRGs, and the *qacE* gene was seen in eight of the plasmids. The most common animal source for IncI plasmids was turkey, with 22 isolates, and 6 of these were in serotype I 4,[5],12:i:-.

#### Other Plasmid Types

Several other plasmid types were identified, including Inc R, A, B/O/K/Z, Q, N, P, F, and Y in descending ARG prevalence, respectively. The 27 IncF plasmids most commonly encoded either no ARGs (10/27) or only aminoglycoside and tetracycline resistance genes (8/27). Only 4/15 IncX plasmids encoded ARGs. Small Col-type plasmids contained few ARGs, with only 7/72 encoding any ARGs, spread widely across different sources (Figure 3). There were 11 IncQ plasmids (aside from those integrated into the chromosome) found among diverse serotypes and sources, nine of which had the genes *sul2*, *aph(6)-Id/aph(3*″*)-Ib*, and *tetA*. There were also three IncN plasmids, two IncA, and one plasmid each of IncP, IncR, IncY, and IncB/O/K/Z.

Twelve plasmids had multiple replicon types, indicating likely recombination between multiple plasmid types. These included IncHI, IncC, IncI, IncN, IncF, IncP, IncQ, and IncX replicons (Supplementary Table 1). All but one of these hybrid plasmids had ARGs for at least three different drug classes.

A total of 44/285 plasmids did not have hits based on PlasmidFinder, indicating a failure of conventional typing techniques to identify them. Even though these plasmids were un-typeable, three contained ARGs (Supplementary Table 1).

Overall, there were 33 *Salmonella* serotypes represented, with some interesting serotype-specific findings. Serotype Agona had *bla*_CMY–__2_ as part of multidrug-resistant IncC plasmids in six of ten isolates. Similarly, MDR IncC plasmids were found in all four *S.* Montevideo and all five *S.* Newport isolates.

Serotype Kentucky also had IncX1 plasmids in ten of eleven isolates, with each of these being from chicken sources. There was one *S.* Kentucky isolate from turkey which did not have this plasmid and it did not possess any ARGs (Supplementary Table 1). Seven *S.* Kentucky isolates also had IncF plasmids with *aph(3*′*)-Ib/aph(6)-Id*, and *tet*(A); this combination of plasmid type and resistance genes was not observed in any other serotypes.

Other serotypes also had specific plasmid/resistance gene combinations. For instance, five of six *S.* Reading isolates had IncQ plasmids with the ARGs *aph(3*′*)-Ib/aph(6)-Id*, *sul2*, and *tet*(A), among only nine total isolates with this combination of plasmid type and resistance genes. All but one of these nine isolates were from turkey sources. Of importance to *Salmonella* virulence is that *spv* operon were found in nine isolates of four serotypes, including five Typhimurium, one I 4,[5],12:i:-, two Dublin, and one Enteritidis. IncF (IncFII(S) subtype) replicons were present in all but one plasmid with *spv* operon, with one missing this replicon likely because it was not fully circularized. IncC and IncHI plasmid types were most strongly associated with ARGs (Table 3).

**FIGURE 4:**
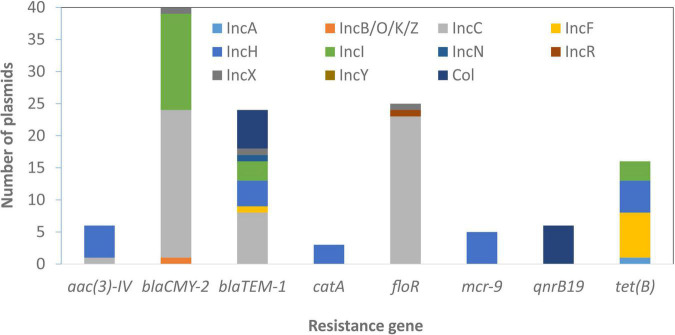
Plasmid-resistance gene associations. The information above relates to plasmids with typing information and does not include those with zero or multiple replicon types.

**FIGURE 5:**
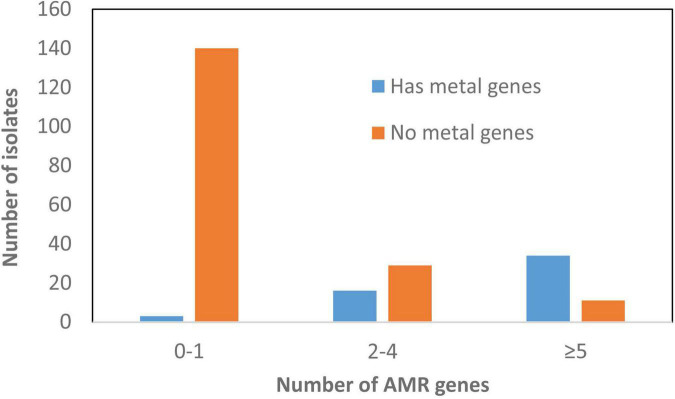
Correlation between presence of antimicrobial resistance genes (ARGs) and heavy metal resistant genes (HMRGs). This depicts information related to plasmids and the presence of these genes.

Some genes that were plasmid-specific include *catA* and *mcr-9* only on IncHI plasmids (Tyson et al., 2020) and *qnrB19* only on Col plasmids. Other genes, such as *bla*_TEM–__1_ and *tet*(B), were more widely distributed across different plasmid types (Figure 4). Overall, there was an association between the presence of ARGs and the presence of HMRGs on the same plasmid (Figure 5).

## Discussion

Here we present the results from long-read sequencing of 134 *Salmonella* genomes, including over two hundred plasmids with circularized sequences. This sequencing effort produced several important findings, including insight into the acquisition of virulence and AMR determinants.

The size of SPIs ranged from 1.7 to 133.3 kb, encoding 1–21 virulence genes. A phylogenetic tree based on the presence and absence of genes in each SPI from our previous study showed that, aside from serotype Choleraesuis, many other serotypes showed great diversity, particularly Newport, Derby, Agona, Anatum, and Typhimurium (Zhao et al., 2020). Some SPIs were more conserved across serotypes, such as SPIs 1-5, which had ≥96% identity and 72% length alignment with the reference SPI in all isolates. Some SPIs had greater variability, such as SPI-6, which in some cases had 97% identity but only 21% length alignment with the reference SPI, although its position relative to other SPIs was consistent in all isolates. Despite the great diversity of SPIs, the virulence genes are highly specific to individual SPIs. The presence of SPIs varied in different serotypes but was largely consistent within individual serotypes (Figure 1). Several publications showed different SPI profiles by using different methods to identify the presence of SPIs (Amavisit et al., 2003; Suez et al., 2013). In this study, a combined method of presence of virulence gene, genomic location and SPI reference structures was used to identify the SPIs.

Rarely, some virulence genes were found in multiple location, for example, *gtr*A genes were shared by both SPI-16 and SPI-17. Since the genomic location of each SPI is conservative, the presence of SPI-16 or SPI-17 were easily decided by this combined method.

In this study, it was found that the relative position of SPIs to each other was generally stable. Even SPIs that are in phylogenetically distant serotypes, such as SPI-18 in Kiambu and Bredeney, had conserved insertion locations. This was true even though the number of SPIs was variable across different serotypes, indicating a key role for the SPIs in the evolution of serotypes.

The presence of additional elements was also able to be easily identified in reference to the SPI positions, as shown in Figure 1. The conserved SPI genomic locations can help to identify large insertions, such as SGIs or other accessory elements. In all cases the combinations of SPIs were consistent within serotypes, which can potentially be used to orient assemblies with short-read data, acting as a scaffold to understand the arrangement of the genome.

Recent research shows that SGI-1 is an integrative mobile element that plays an important role in introducing antibiotic resistance in various Gram-negative bacteria, including *S. enterica, Proteus mirabilis*, and *Acinetobacter baumannii* (Cummins et al., 2020). We identified twelve isolates with SGI-1 sequences, either by homology to the reference SGI-1 sequence or from insertions in the same region. All 12 SGIs were similar to SGIs in *Proteus mirabilis*, but their close relatives also included sequences from *Citrobacter* and *Enterobacter*. This finding further helped us to understand how these genomic islands were horizontally acquired (Table 1).

We had other novel findings, including an SGI-1 sequence in serotype Alachua and large SGI-1s of different origin in serotypes Senftenberg, and Saintpaul. Those SGI-1 had almost no homology to previously reported SGI-1. The great diversity made it impossible to name the variants alphabetically as typical approach. In this study we also resolve the issue associated with the naming of SGI sequences by proposing a new approach based on their relative position in the genome. For instance, SGI-0 and SGI-2 (Levings et al., 2008; de Curraize et al., 2020) as previously name can all named as SGI-1 based on their consistent positions with other SGI-1 variants. An additional example of SGI diversity is shown by a novel SGI island containing HMRGs in *S.* Alachua. Even though it has limited homology with a previously reported SPI-4 (95% identity and 40% length), it was named as SGI-4 variant because of its genomic location. By naming SGIs based on location, we hope for a streamlined process for SGI nomenclature in future work as diverse SGI sequences are identified. It can help to identify the potential new variants. It would be interesting to further investigate the prevalence and distribution of SGIs in *Salmonella* isolated from other sources, including from human and sick animals. In this study, we also found ARGs and HMRGs on many chromosomal sequences with 100% homology to plasmids, indicating fragment of plasmid integration into the chromosome (Table 2). In fourteen isolates of *S.* I 4,[5],12:i:-, MDR IncQ plasmids were inserted into the same location in the chromosome (Figure 2). This monophasic serotype often results from different insertions, deletions, or other disruptions of the *fljB* gene in serotype Typhimurium (Yang et al., 2015). We believe our results are the first to identify insertion of IncQ at this site and may have resulted in *S.* Typhimurium conversion to serotype I 4,[5],12:i:-.

As expected, most AMR was mediated by plasmids. A total of 147 plasmids had one or more ARGs compared to only 74 chromosomes, some of which carried integrated plasmids as well as genomic islands. Although there were certain plasmid type/resistance gene associations found only in particular serotypes, most were not typically source or serotype-specific and carried diverse plasmid types linked with different AMR genes and HMRGs. In addition, we found that 60% MDR (>3 AMRGs) strains also carried >3 HMRGs, including those conferring resistance to copper, gold, mercury, silver, arsenic, and tellurium (Supplementary Table 1 and Figure 5). This co-existing of AMRs and HMRs is of interest as the presence of any of these metals in food animal production have the potential to co-select for AMR. This is of particular significance as these congregated HMRGs were found on newly discovered SGI-4 and plasmids conferring resistance to three or more antimicrobial classes (Figure 5).

There were some limitations in this study. Only 134 *Salmonella* isolates were sequenced, they were exclusively from food animals and retail meats, and the isolates were not randomly chosen. As a result, findings from this study may not be broadly applicable to all *Salmonella* serotypes or genomes from different animals, foods, or environments. In addition, we focused our sequencing on multidrug-resistant isolates, so some plasmids found to be frequently associated with AMR may have lower associations in a broader context of *Salmonella* serotype or genomic diversity. Also, our work highlights a drawback of using incompatibility typing to identify plasmid types, as some plasmids often have either multiple replicon sequences or none. Furthermore, isolates with the pESI plasmid in *S.* Infantis were only identified with IncF replicons, despite the fact that this plasmid resulted from a combination of multiple plasmid types (Tyson et al., 2021). Given these challenges, alternative approaches such as the use of Plasmid Taxonomic Units could help address at least some of these issues (Redondo-Salvo et al., 2020). Despite the limitations, this study represents perhaps the largest collection of closed *Salmonella* genomes reported to date and advances our understanding of *Salmonella* genomics including its genomic plasticity and evolution.

Future work to evaluate the applicability of long-read data to short-read datasets, including the use of reference-assisted assemblies, will increase the level of detail in genome-based AMR surveillance such as that done in NARMS and other national surveillance programs. Greater efforts to close *Salmonella* genomes also will help improve our understanding of genomic plasticity, evolution, and virulence. This work will help refine risk assessments by revealing the associations of resistance and virulence on mobile DNA elements, making it possible to be more precise in targeted interventions to limit the spread of the most problematic *Salmonella* serovars, including those less likely to respond to antimicrobial therapy.

## Data Availability Statement

The datasets presented in this study can be found in online repositories. The names of the repository/repositories and accession number(s) can be found in the article/Supplementary Material.

## Author Contributions

CL, GHT, and SZ: conceptualization of ideas, validation, formal analysis, investigation, and writing—original draft preparation. CL, GHT, C-HH, and SZ: methodology. CL, C-HH, and ES: software. ES, GET, UD, and PM: resources. CL and T-TT: data curation. CL, GHT, C-HH, LH, ES, T-TT, GET, UD, PM, and SZ: writing—review and editing. CL, GHT, and C-HH: visualization. ES, PM, and SZ: supervision. ES and PM: project administration and funding acquisition. All authors have read and agreed to the published version of the manuscript.

## Author Disclaimer

The views expressed in this article are those of the authors and do not necessarily reflect the official policy of the Department of Health and Human Services (DHHS), the United States Department of Agriculture (USDA), the United States Food and Drug Administration (FDA), or the United States Government. Reference to any commercial materials, equipment, or process does not in any way constitute approval, endorsement, or recommendation by the FDA, USDA, or the United States Government.

## Conflict of Interest

The authors declare that the research was conducted in the absence of any commercial or financial relationships that could be construed as a potential conflict of interest.

## Publisher’s Note

All claims expressed in this article are solely those of the authors and do not necessarily represent those of their affiliated organizations, or those of the publisher, the editors and the reviewers. Any product that may be evaluated in this article, or claim that may be made by its manufacturer, is not guaranteed or endorsed by the publisher.
